# The Impacts of Inflammatory and Autoimmune Conditions on the Endometrium and Reproductive Outcomes

**DOI:** 10.3390/jcm13133724

**Published:** 2024-06-26

**Authors:** Isabel Cuadrado-Torroglosa, Juan A. García-Velasco, Diana Alecsandru

**Affiliations:** 1 IVIRMA Global Research Alliance, IVI Foundation, Instituto de Investigación Sanitaria La Fe (IIS La Fe), Avenida Fernando Abril Martorell, 106, Torre A, Planta 1a, 46026 Valencia, Spain; isabel.cuadrado@ivirma.com (I.C.-T.); juan.garcia.velasco@ivirma.com (J.A.G.-V.); 2 IVIRMA Global Research Alliance, IVIRMA Madrid, Av. del Talgo, 68, 28023 Madrid, Spain; 3 Department of Obstetrics and Gynaecology, Rey Juan Carlos University, Av. de Atenas, s/n, 28922 Alcorcón, Spain

**Keywords:** chronic inflammation, autoimmune disease, endometrium, decidualization, antiphospholipid syndrome, endometritis, thyroid disfunction, celiac disease, diabetes

## Abstract

**Background**: A healthy pregnancy begins with an adequate endometrial state, even before the arrival of a blastocyst. Proper endometrial priming and the development of a tolerogenic decidua are key steps in creating the perfect environment for implantation and pregnancy. In these processes, the involvement of the maternal immune system seems to be of great relevance, modulating the different decidual immune populations to prepare the endometrium for a potential pregnancy. However, certain local pathologies of an inflammatory and autoimmune nature appear to have a direct impact on these phenomena, thus altering patients’ reproductive outcomes. **Methods**: This literature review analyzes original articles, reviews, systematic reviews, and meta-analyses published between 1990 and 2024, concerning the impact of different inflammatory and autoimmune conditions on endometrial status and fertility. The included papers were obtained from Medline (Pubmed) and the Cochrane library. **Results**: There is evidence that endometriosis, adenomyosis, and chronic endometritis, through the promotion of a chronic inflammatory environment, are capable of altering endometrial immune populations, and, thus, processes essential for early pregnancy. Among other effects, these conditions have been linked to impaired decidualization, alterations in progesterone responsiveness, and hindered placentation. Similarly, antiphospholipid syndrome (APS), thyroid dysfunction, diabetes, and other pathologies related to glucose and gluten metabolism, due to their autoimmune nature, also appear to have a local impact on the uterine environment, affecting reproductive success through different mechanisms, including altered hormonal response and, again, impaired decidualization. **Conclusions**: The management of inflammatory and autoimmune diseases in assisted reproduction patients is gaining importance due to their direct impact on the endometrium. It is necessary to follow current expert recommendations and established therapeutic approaches in order to improve patients’ prospects, ranging from antibiotic treatment in chronic endometritis to heparin and aspirin in APS, as well as hormonal treatments for endometriosis/adenomyosis or a gluten-free diet in celiac disease. All of them and the rest of the therapeutic perspectives, both current and under investigation, are presented throughout this work, assessing the possible improvements for reproductive outcomes.

## 1. Introduction

A successful pregnancy depends on both a competent embryo and a receptive endometrium. This binomial must work together to ensure an adequate coexistence of both individuals during gestation, while favoring embryo development until birth. Today, advances in IVF techniques, including pre-implantation genetic testing, time-lapse incubation, or even the use of artificial intelligence for embryo selection, have drastically improved reproductive outcomes [1,2,3]. 

In this scenario, many clinicians and researchers focused their attention on the embryonic factor, sometimes underestimating the importance of the endometrial status of patients. Today, however, many studies have demonstrated the essential involvement of the endometrium in achieving a healthy pregnancy, with several researchers even proposing that “*the primary driver of pregnancy health is the quality of the soil, not the seed*” [4]. Although not all authors agree with this statement, research on the potential relevance of the endometrium to implantation and pregnancy has spread over the last decades.

Indeed, numerous investigations have highlighted the impact that endometrial priming, or decidualization, can have on reproductive success. Accordingly, proper decidualization, involving modifications at all biological levels, is required to obtain decidual endometrial cells and, with them, an endometrium conducive to pregnancy [4]. Situations of impaired decidualization have been related to undesired reproductive outcomes, such as intrauterine growth restriction [5], pre-eclampsia [6], recurrent pregnancy loss (RPL), or recurrent implantation failure (RIF) [7], demonstrating the actual importance of endometrial priming. 

Interestingly, some of the most relevant modifications during decidualization are immunological in nature. In this regard, and in response to progesterone, decidualized stromal cells secrete a significant number of cytokines, which, in turn, favors the recruitment and proliferation of leukocytes towards the endometrium [5]. Thus, the uterine environment is populated with, mainly, uterine natural killer (uNK) cells, macrophages, and T cells, each of which will play relevant roles for pregnancy [4,5].

These immune changes are not minor; in fact, the endometrial environment completely modifies its immune subsets throughout the secretory phase, while modulating the expression of certain genes, many of them related to inflammation and immune regulation [8]. In this way, a controlled balance between pro- and anti-inflammatory environments is promoted in order to support implantation and pregnancy. According to recent research, the expected pro-inflammatory environment of the peri-implantation period should change rapidly, shortly after implantation, to an anti-inflammatory milieu, which is essential for placentation and fetal development [9,10,11]. 

These hypotheses are consistent with publications observing such an immune adaptation in healthy pregnancies, in which proinflammatory and cytotoxic responses are inhibited. On the contrary, scenarios of unexplained recurrent miscarriages and implantation failure have been related by some authors to uncontrolled immune scenarios, with marked increases in certain pro-inflammatory immune cell populations and cytokines [9,10,11].

In fact, it has been demonstrated that a progesterone-dependent immunomodulation should occur during decidualization, and extends throughout the gestational period, ensuring embryonic tolerance, maintaining the immune response to pathogens, and promoting the appropriate environment for proper placentation and embryonic development. It is not surprising, therefore, that situations capable of altering these immune adaptations may be associated with poorer reproductive outcome [9]. 

In this regard, specific diseases, such as endometriosis, adenomyosis, and chronic endometritis, very well studied in the context of reproduction, have recently attracted attention because of their link with chronic endometrial inflammation. Indeed, all these conditions appear to involve significant alterations in immune cell populations and, consequently, in cytokine production, favoring pro-inflammatory states that, per se, could be hindering pregnancy, along with the other mechanisms by which these diseases can directly impact fertility [12].

Similar scenarios can occur in relation to antiphospholipid syndrome, thyroid disfunction, diabetes, or celiac disease, conditions commonly faced by physicians in the ART setting and that share a common feature: their autoimmune nature. Many authors have pointed to the immune hyperactivation, typical of these systemic illnesses, as a possible cause for the poorer reproductive success registered in these patients, suggesting that a local impact on the endometrium may exist in these cases [13,14,15].

Indeed, all of the disorders mentioned so far seem to be able to impact the endometrium and alter immune cell populations, by one mechanism or another, always promoting pro-inflammatory states that potentially hinder immune tolerance and an adequate pathway to implantation and free-risk gestation [14,16,17]. However, the associated immunological mechanisms by which these diseases may impact reproduction are not yet fully understood and are often overlooked when treating patients. It is necessary to fully describe the specific immunological effects that these pathologies can cause at the uterine level and, consequently, on reproductive success, in order to guide clinicians in the treatment of these couples, especially when dealing with idiopathic infertility scenarios [14,16]. 

Therefore, the aim of this review is to evaluate the endometrial repercussions, related to inflammation and immune hyperactivity, that have been described to date, associated with these inflammatory and autoimmune pathologies. Likewise, and in accordance with the latest publications, we will review the current medical advice for these patients, addressing the tests and therapies recommended for these conditions and their use in the ART setting, particularly in the case of infertility of unknown etiology. Finally, we will highlight the latest advances in the field aimed at improving the prognosis and reproductive expectancy of patients facing any of these conditions.

## 2. Pro-Inflammatory Endometrial Pathologies

### 2.1. Endometriosis

Endometriosis is a common estrogen-dependent inflammatory pathology that has been estimated to affect 30–50% of women with infertility. With its pathogenesis not being completely understood, this disease is characterized by the presence of endometrial-like tissue outside its normal location [18,19]. Research on endometriosis soon demonstrated the involvement of the immune system in the development of this condition, playing a key role in its pathophysiology and symptomatology [19,20].

Indeed, a transcriptomic analysis of endometrial samples revealed that women with endometriosis have altered steroid hormone signaling and up-regulation of pathways related to lymphocyte activation, antigen presentation, cytokine production, and inflammation [21,22]. Alterations in decidualization related to this disease have also been recorded, showing a direct involvement of endometriosis in this priming, which, as explained above, is essential for implantation and the onset of a healthy pregnancy [23]. 

In general, endometriosis research over the past decades has come to similar conclusions: an altered pro-inflammatory environment in the endometrium, along with various modifications in non-immune cells that contribute to the development and symptomatology of this condition. Some of the immune subsets that have been reported to be altered in patients with endometriosis include macrophages, dendritic cells (DCs), natural killer (NK) cells, and T cells [19]. 

In this regard, a decrease in the M2 phenotype (anti-inflammatory) of macrophages has been observed in women with endometriosis compared to controls [24]. This is the predominant phenotype in healthy endometrium. It has, therefore, been suggested that a pro-inflammatory environment may be promoted in the endometrium of women with this pathology, which, in turn, may result in the secretion of numerous pro-inflammatory cytokines and angiogenic and growth factors, unfavorable for embryo implantation [19,25].

**Figure 1:**
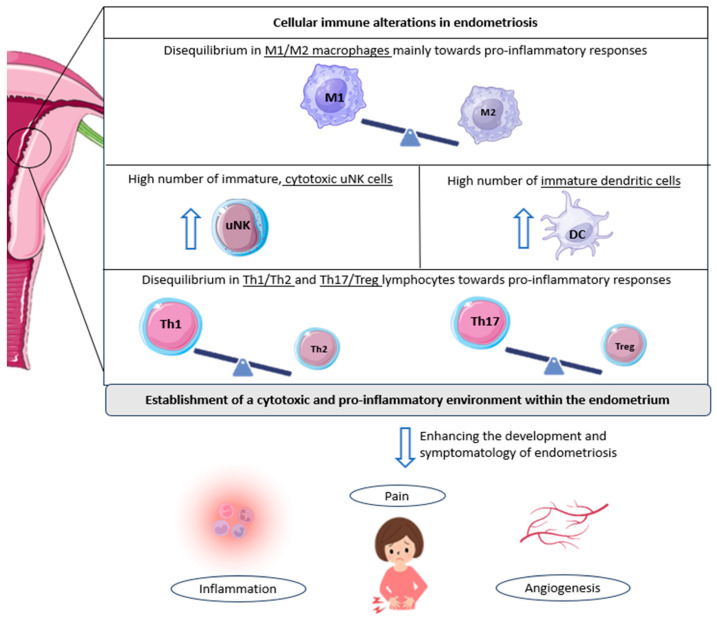
Cellular immunological alterations and associated symptomatology in patients with endometriosis. The underlined text indicates the cell populations altered in endometriosis; in other words, the imbalance of M1/M2 macrophages, Th1/Th2 lymphocytes, and Th17/Tregs reported in patients with endometriosis, along with the observed increase in other immune cell populations, such as cytotoxic NK cells and immature dendritic cells. All these cellular alterations favor the development and associated symptomatology of endometriosis, including inflammation, pelvic pain, and angiogenesis, further promoting endometriotic lesions. DC: dendritic cell; Th: T helper cell; Treg: regulatory T cell; uNK: uterine natural killer cell. Original, created with Biorender.com and SMART.servier.com (accessed on 22 May 2024).

Further research corroborated these ideas, finding an increased presence of M1 macrophages (pro-inflammatory) in women with endometriosis (Figure 1) [26]. Interestingly, some authors observed that a trend towards a progressive decrease in M1 macrophages exists from stage I to stage IV disease; in contrast, M2 macrophages progressively increase compared to M1 macrophages from stage I to stage IV. This phenomenon may play a role in the pro-inflammatory environment characteristic of early-stage disease, which then gives way to the profibrotic activity of advanced stages [27].

The repercussions that endometrial immunological alterations have on fertility are multiple, being related, among others, to ectopic pregnancy or spontaneous abortion [19,28]. However, in the particular case of macrophages, it has been suggested that this aberrant behavior and their increase are involved in the pain characteristic of endometriosis [19]. Macrophages are able to secrete cytokines, prostaglandins, and neurotrophins, which can activate nerve fibers in different tissues, thus causing pain in these regions [29,30]. Nevertheless, more research is needed to fully clarify this association.

Likewise, a similar connection has been established with endometriosis-related pain and dendritic cells. An increase in immature DCs has been reported in endometriotic lesions compared to controls (Figure 1). This fact, together with the knowledge that DCs are able to promote angiogenesis and increase pain perception due to the down-regulation of opioid receptors, leads researchers to focus on these cells when studying the symptomatology of the disease. In addition, the lack of maturation of these cells in patients with endometriosis suggests an impairment of their normal functions, which probably leads to the altered shedding of endometrial cells during menstruation, further complicating the situation in the endometrium [19,31].

A comparable scenario can be observed with regard to uterine natural killer cells. Indeed, a high number of immature uNK cells has again been observed in patients with endometriosis [32]. Moreover, these same authors also found an increased expression of cytotoxicity markers in women suffering from this condition, who were also diagnosed as infertile or had a history of RPL [32]. Given that these cells tend to show low cytotoxic activity in the healthy uterus, this aberrant cell behavior could serve as an indicator of endometrial-related fertility problems, rendering the endometrium in a state not conducive to pregnancy (Figure 1) [19]. 

In addition, one of the most relevant uNK receptors in the context of reproduction and maternal–fetal crosstalk, the killer immunoglobulin-like receptor (KIR), shows alterations in cases of endometriosis. These receptors modulate the behavior of uNKs and have been linked to proper decidualization and the promotion of embryo invasion and placentation [33]. A higher expression of inhibitory KIRs and a low presence of activating KIRs have been described in the endometrium of women suffering from this pathology [34,35]. An inhibitory pattern is thus established, associated with endometriosis, with increased cytotoxicity by uNKs, while impairing the role of these cells in supporting embryo implantation [17,19]. 

Nevertheless, one of the main immune populations related to endometriosis and its progression are T cells. Several subsets exist within the uterus, with T helper cells (Th1, Th2, and Th17) and regulatory T cells (Tregs) being the more relevant in this regard [19]. Interestingly, some authors have reported that Th1 cells (pro-inflammatory) are significantly more abundant in patients with endometriosis compared with healthy women (Figure 1) [36]. In addition, increased levels of specific Th1 cytokines, tumor necrosis factor (TNF)-α, and IL-2 have been found in women suffering this disease, with a high presence in cases of deep infiltrating endometriosis, again linking this inflammatory pattern to the severity of the condition [37].

Likewise, the involvement of Th17 cells in endometriosis has been extensively studied. These cells are also responsible for the development of pro-inflammatory responses and are directly implicated in the recruitment of immune cells to sites of inflammation [19]. In women with endometriosis, Treg/Th17 imbalances have been described, with an increased Th17 cell count, compared to individuals without the disease. In addition, the abundance of Th17 cells seems to be significantly higher in the peritoneal fluid of patients with severe endometriosis compared to mild cases, supporting a role for these cells in the progression of this condition [38]. Moreover, some authors have found a link between these immunological imbalances and undesired outcomes, as defective endometrial receptivity or recurrent miscarriage [39,40].

In conclusion, it is now well-known that a favored cytotoxic and pro-inflammatory environment is characteristic of endometriosis. Likewise, ‘neuro-angiogenesis’ (the growth of new blood vessels and nerve fibers) also appears to be enhanced in these patients, contributing to the progression of endometriotic lesions and pain [12,19,41]. Endometrial immune cells are modulated differently in women with this pathology, further exacerbating an inflamed, per se, endometrium. An increased number of activated macrophages and other immune populations, and differences in the cytokine/chemokine profile are representative of women suffering from endometriosis [42]. 

Accordingly, it seems that the most important factor in the endometrial alterations observed in patients with endometriosis is associated with inflammation-mediated histological changes. Indeed, alterations in the endometrial response to hormones seem to occur due to this condition, consequently shaping endometrial receptivity [12,43]. These findings are supported by research pointing to inflammation as the main mechanism by which progesterone resistance and estrogen receptor dominance occur in patients with endometriosis [12,44,45]. 

Inflammation, therefore, is considered by many experts in the field as the ultimate cause of the impaired implantation sometimes faced by these patients, due to its direct impact on decidualization and endometrial receptivity, through a functional dysregulation of steroid hormone signaling [12,43]. However, caution should be exercised in this regard, as some authors report no changes in endometrial receptivity associated with this pathology, in oocyte-donation cycles, [46], while others observe only a marginal effect that may contribute to infertility in these patients [47].

Another interesting aspect of endometriosis is its link with autoimmune pathologies. Indeed, several epidemiological studies have observed significant associations between the occurrence of endometriosis and certain diseases, such as thyroid dysfunction, both of which are able to affect reproductive outcomes [48,49]. Although this topic will be discussed later in this review, it is important to address such relationships in order to understand the full potential impact of this pro-inflammatory condition on fertility, with studies even finding a differential gene expression in steroid and thyroid hormone signaling/metabolism in women with endometriosis [22].

Treatment options for women suffering from endometriosis would vary depending on the severity of symptoms, the extent and location of the disease, the desire to become pregnant, and the age of the patient. The two approaches currently used are medication and surgery, either individually or in combination. Hormonal treatments used in the management of endometriosis aim to inhibit ovulation and menstruation, and reduce the state of chronic inflammation that accompanies the disease [50,51].

The options include hyperprogestogenic therapy (combined oral contraceptives and progestins), hypoestrogenic therapy (gonadotropin-releasing hormone—GnRH agonists), and hyperandrogenic therapy (danazol or gestrinone). All these treatments focus on inhibiting ovarian estrogen production, and, with it, ovulation and decidualization, a situation that favors the reduction and atrophy of the endometriotic implants [51,52]. Interestingly, other options currently exist that act in blocking estrogen production, not in the ovary, but directly in the endometriotic lesions. They are aromatase inhibitors. This therapy is highly specific, acts locally, and is able to reduce the lesions, while decreasing pelvic pain. The most commonly used in this regard are third-generation drugs such as anastrozole and letrozole [51,53]. 

However, as endometriosis is now considered a chronic inflammatory disease, several lines of research have flourished in this regard, to develop specific treatments for this condition. Until now, medications such as paracetamol and non-steroidal anti-inflammatory drugs (NSAIDs) were the only treatments with these characteristics, used to relieve patients’ pain. However, the importance of the inflammatory factor in endometriosis has led to research into other promising new therapies [41]. 

Some of them are based on the increased production of pro-inflammatory cytokines recorded in women with endometriosis, such as IL-1, IL-6, IL-8, IL-33, TNF, or insulin-like growth factor 1 (ILGF-1) [41,54]. These molecules and their receptors, therefore, represent interesting targets for monoclonal antibody therapies. Indeed, several clinical trials have emerged, testing some of these new approaches. Examples of these investigational treatments include: a monoclonal antibody directed against IL-33 (MT2290), with favorable results in the preclinical phase; an IL-1 receptor antagonist (NCT03991520), used for the treatment of other inflammatory diseases, such as rheumatoid arthritis [41]; or anti-TNF therapy (infliximab), which, although initially promising, did not yielded positive clinical outcomes [55]. 

Other current research is based on the involvement of prostaglandins in the establishment of a pro-inflammatory state. Despite relevant side effects shown in clinical trials testing certain therapies targeting the prostaglandin biosynthesis pathway (COX-2 inhibitors and AKR1C3 enzyme inhibitors) [41,56], some promising alternatives are emerging, based on prostaglandin receptor agonists or antagonists (EP1–EP4) [41,57]. Likewise, other possible approaches which are currently being tested in animal models, include novel estrogen receptor ligands [58] and certain anti-helminth drugs, with interesting inhibitory effects on macrophage-induced inflammation [59]. 

The increasingly widespread understanding of endometriosis as an inflammatory disease is allowing the emergence of all these new treatments, which aim to control this disease in a highly specific way, decreasing the effects of inflammation on the endometrium and, thus, the consequences of this pathology. Systemic and local hormonal treatments and NSAIDs constitute the therapeutic approaches currently available for ART patients with endometriosis; however, research continues to find increasingly innovative and targeted treatments. The study of this condition from the immunological point of view and the management of patients from these new perspectives will undoubtedly improve the treatment of endometriosis and, in the future, the clinical outlook for women suffering from it [12,17,41,51].

### 2.2. Adenomyosis

Adenomyosis is a reproductive pathology characterized by the existence of ectopic endometrium, along with endometrial glands and stroma, which develops in the myometrium [12]. It is known as a benign condition with symptoms that includes dysmenorrhea, menorrhagia, abnormal uterine bleeding, infertility, and chronic pelvic pain [60]. This pathology has been pointed out by researchers as a possible cause of infertility and an altered obstetric outcome, increasing the risk of miscarriages while reducing the likelihood of clinical pregnancy and implantation. This negative impact on fertility is attributed to two main mechanisms: a favored pro-inflammatory state and impaired uterine contractility [12,52]. 

Indeed, some research reported different cytokine profiles in the endometrium of women with and without adenomyosis, with significantly higher levels of IL-6, IFN-γ, and monocyte chemoattractant protein-1 (MCP-1) in adenomyosis patients. A reduction in IL-10 (anti-inflammatory cytokine) was also observed in these women. It was, therefore, concluded that adenomyosis seems to favor host immune responses, leading to both hormonal and cell-mediated inflammation [16,61].

Similar to endometriosis, a proinflammatory state is created in the eutopic and ectopic endometrium of women with this disease, altering the presence and activity of different populations of immunocompetent cells. In fact, these authors also observed a significant increase in macrophages in patients with adenomyosis, which, in turn, are able to promote the local production of, among others, cytotoxic cytokines and prostaglandins. This scenario would lead to an increase in myometrial contractility, further complicating embryo implantation [61].

These results are consistent with those of other researchers who, along with the increased presence of macrophages associated with adenomyosis, also observed an elevated density of NK cells in these patients [62]. The increased presence of pro-inflammatory cytokines associated with adenomyosis suggests the occurrence of some imbalance between M1/M2 macrophages and Th1/Th2 cells (both towards M1 and Th1 responses) [16]. Furthermore, some research has reported imbalances in the Th17/Treg cell ratio in patients with this condition, with a marked increase in the Th17 population [63]. 

This pro-inflammatory environment promotes drastic alterations at the endometrial level, with endocrine disruptions similar to those observed in patients with endometriosis. In fact, in these cases, there seems to be a low responsiveness to progesterone, which affects the endometrial dynamics entirely and alters the decidualization process [64]. Indeed, some authors state that scenarios of persistent inflammation may represent a cause of decidualization dysfunction [64,65]

Research on the impact of adenomyosis on decidualization includes a recent study showing the altered expression of genes involved in endometrial priming and a higher rate of non-receptive endometrial states in women with this disease. Moreover, the adjustment of progesterone administration prior to the personalized embryo transfer did not improve the reproductive outcome in these patients. According to the authors, these results point to the existence of other molecular mechanisms beyond progesterone regulation, involved in adenomyosis-mediated implantation failure [66].

The treatment and research of adenomyosis is complicated by the fact that this disease may coexist with other gynecological comorbidities, like endometriosis or uterine fibroids [60,67]. However, there are numerous projects in this field, which are trying to develop new therapies for this pathology, while various hormonal and non-hormonal treatments are used *off-label* to alleviate the symptoms and prospects of adenomyosis patients [60]. 

One of the most commonly used drugs, especially considering the pro-inflammatory factor associated with adenomyosis, are NSAIDs, also used in endometriosis. They are a common treatment used to alleviate the symptoms of dysmenorrhea or pelvic pain related to this pathology [60]. Likewise, the use of progestins, such as Levonorgestrel, seems to be useful in the management of adenomyosis, as it has a direct and local effect on adenomyotic foci, while enhancing decidualization and mitigating endometrial alterations [60].

Other current approaches include combined oral contraceptives, that lead to decidualization and the subsequent atrophy of adenomyotic lesions, while, at the same time, seeming to exert an anti-inflammatory effect on adenomyotic foci [60,68]. Similarly, and as in the case of endometriosis, the use of GnRH agonists and antagonists is being explored for adenomyosis patients [60,69], as is the employment of selective progesterone receptor modulators (SPRMs), which have been shown, among other effects, to reduce pain and inhibit inflammation. Interestingly, SPRMs are able to decrease the secretion of pro-inflammatory cytokines like IL-6 and TNF-α, limiting the infiltration of specific immune cells at the endometrial level [70]. 

Aromatase inhibitors also appear to exert some anti-inflammatory activity in the endometrium. Their mechanism is based on the abnormal expression of aromatase P450, observed in women with pathologies such as endometriosis or adenomyosis, which, in turn, is enhanced by prostaglandin E2. This leads to an elevated estrogen production and an increased expression of this prostaglandin, which further promotes inflammation [71]. Therefore, the use of these inhibitors is an option currently being considered for patients suffering from these types of endometrial pathologies, especially in cases of severe adenomyosis [60,69]. 

However, as already mentioned, the therapies presented here are typically used *off-label* in the context of ART, as there is no authorized medical treatment for adenomyosis, and the evidence available for determining the preferred medical approach is significantly limited. This arises partly due to the complexity of the diagnosis and partly due to the prevalence of concurrent gynecological conditions [60]. Research into the pathophysiology of this disease, and the in-depth study of the inflammatory factor in this regard, could improve the current situation of patients, bringing us closer to a complete understanding of the endometrial and, consequently, reproductive aspect of this pathology.

### 2.3. Chronic Endometritis

One of the most characteristic conditions that come to mind when we think of endometrial inflammation is undoubtedly chronic endometritis (CE). This pathology, in fact, is defined as a continuous and mild inflammation of the endometrial lining, presumably due to a microbial infection in the uterine cavity [12,72]. To date, CE has been considered a benign disease, commonly overlooked by patients and ignored by physicians, given its mild symptomatology. 

However, the impact that CE can have on patients’ reproductive outcomes is causing this disease to gain attention in recent years [72]. Indeed, CE is diagnosed in 28% of infertile women of unknown etiology, and a link between this condition and poorer reproductive outcomes has been observed by some authors, registering a higher incidence of CE in women with a history of implantation failure and RPL. These findings point to a relevant clinical impact of chronic endometritis in reproduction and the need to study and treat this disease in the ART setting [12,72,73]. 

**Figure 2:**
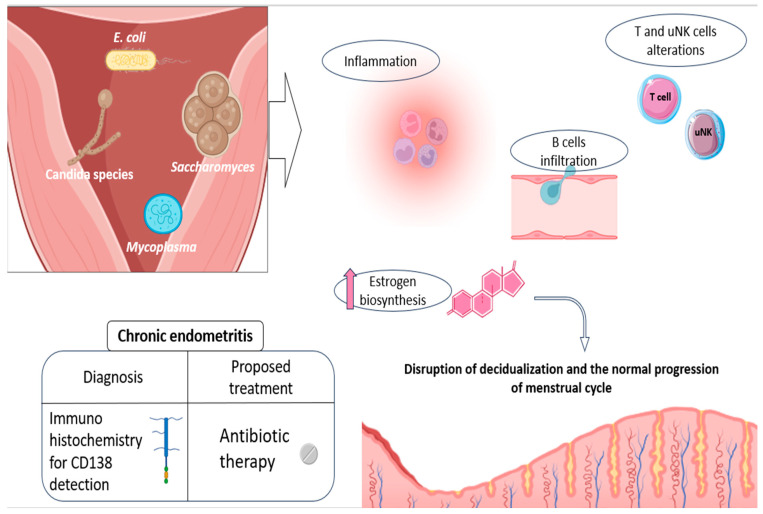
Etiology and physiopathology of CE, along with the current advice for its diagnosis and treatment. Some of the main pathogens related to CE development are shown, as well as the main endometrial alterations they promote: disturbances in NK and T-cell populations, promotion of a pro-inflammatory environment, and increased B-cell recruitment and infiltration, as well as hormonal alterations, which actively contribute to the impaired decidualization. The currently recommended diagnosis (CD138 detection) and treatment (oral antibiotics) are also shown in the figure. Original, created with Biorender.com and SMART.servier.com (accessed on 22 May 2024).

In general, micro-organisms associated with the development of CE include a number of common bacteria, both streptococcus and staphylococcus species (*Escherichia coli*, *Enterococcus faecalis*, etc.), mycoplasma/ureaplasma species (*Mycoplasma genitalium*, *Ureaplasma urealyticum*, etc.), proteus species (*Klebsiella pneumoniae*, *Gardnerella vaginalis*, *Corynebacterium*, etc.), and certain yeasts (*Saccharomyces cerevisiae* and candida species) (Figure 2) [72,74]. An altered presence of some species of anaerobic lactobacilli has also been linked to this condition, although with some controversy in existing studies [72].

Consistent with its nature as an infectious disease, CE leads to the establishment of a pro-inflammatory endometrial state, entailing significant alterations in uterine immune populations. In other words, the presence of CE-related micro-organisms is thought to enhance the immune response within the endometrium, completely altering its normal cellular environment and cyclic dynamics [12,72]. In this regard, increased B-cell infiltration has been reported in patients with this disease, with this cell population even penetrating the glandular epithelial zones and further invading the gland lumina [72,75]. These cellular patterns can be explained by the aberrant expression of numerous adhesion molecules and chemokines involved in B-cell extravasation and migration (CD62E, CXCL1, and CXCL13), described in CE patients [75], along with other associated cytokines such as IL-6 [76].

In addition, a lower percentage of CD16^negative^ CD56^positive/bright^ natural killer cells and an increase in T cells were observed in CE patients, compared to the healthy endometrium. All of this reveals significant alterations in the proportions of endometrial immune cells and, consequently, in the reproductive functions in which they may be involved (Figure 2) [72,77]. Likewise, several cytokines of a pro-inflammatory nature, like IL-1β and TNF-α, were also reported to be elevated in the uterine cavity of CE patients [76]. Some of these secreted products seem to be able to alter the endocrine endometrial environment, by favoring, for instance, estrogen biosynthesis, potentially disrupting the normal progression of menstrual cycle (Figure 2) [78]. 

In line with these statements, some authors argue that a delay in endometrial differentiation in the mid-secretory phase is considered common in cases of EC, with patients presenting some form of “out-of-phase” endometrial morphology [79]. These women exhibit an altered pattern of genetic expression, with a marked down-regulation of some genes potentially associated with receptivity and decidualization (IL11, CCL4, IGF1, CASP8, PRL, and IGFBP1) [80,81]. These results suggest that a disruption in the hormonal responsiveness of the endometrium may be occurring in women suffering from this condition, which would hinder the cellular modifications necessary for endometrial receptivity. This scenario of progesterone resistance is similar to those already discussed in the case of endometriosis and adenomyosis, leading to an impaired endometrial receptivity, that contributes to the poorer reproductive outcomes faced by these patients [72]. 

Currently, the detection, through immunohistochemistry, of the plasmacyte marker CD138 (syndecan-1) is considered the most reliable and efficient diagnostic approach for CE [82]. Studies have also observed this CE marker in patients with certain endometrial lesions, such as polyps. According to the authors, this association suggests possible etiopathogenetic links between chronic inflammation and endometrial polyps [83,84,85]. Interestingly, after a hysteroscopic polypectomy, CE also disappeared in these patients, consistent with the presence of CD138. This points to the associations between inflammatory diseases (CE) and impaired endometrial states, highlighting the importance of taking into account the existence of endometrial lesions when deciding the best therapeutic approach for these women [85]. 

Beyond these associations, and in relation to CE treatment, accumulating evidence suggests that the use of antibiotics potentially improves the reproductive outcome in infertile women diagnosed with CE, with some studies reporting the live birth rate (LBR) increasing from 7% to 56%, before and after antibiotic treatment [73]. Therefore, and although prospective randomized controlled trials are still required to verify these conclusions, the current studies point to oral antibiotics as the recommended therapy for CE patients before undergoing IVF treatment [12,72] (Figure 2). The drug of choice has commonly been doxycycline, due to its broad spectrum in the treatment of infections [72]. However, recommendations for different antibiotic regimens have now been established, for the treatment of CE in women with a history of RIF [86]. 

The adequate management of CE is necessary, as not only has this condition been related to poorer reproductive outcomes, but some authors have also found a link between this illness and a certain pathology affecting the placenta, known as chronic deciduitis [72,87]. This is a pro-inflammatory scenario related to obstetric complications, such as preterm labor or neonatal periventricular leukomalacia/cerebral palsy. This condition is characterized by a high infiltration of B cells, and certain epidemiologic studies support that chronic deciduitis originates in preconceptional CE [72,87,88]. Such implications during implantation, but also throughout placentation and the establishment of pregnancy, are an indication of the true relevance of the CE and the inflammatory factor in the endometrium, which should be understood and managed for any woman seeking pregnancy [12,72].

## 3. Autoimmunity: Impact on Endometrium

### 3.1. Antiphospholipid Syndrome

Antiphospholipid syndrome (APS) is a systemic autoimmune disease with symptoms that include arterial, venous, or small vessel thrombosis, thrombocytopenia, miscarriages, or obstetric complications. This scenario is normally associated with the presence of antiphospholipid antibodies (APAs), the most common being anticardiolipin antibodies (aCLs), anti-beta 2 glycoprotein-I (aβ2GPI), and lupus anticoagulant (LAC) [89,90]. 

Nowadays, there is increasing evidence that thrombophilias are associated with numerous obstetric complications, due to the impact that these conditions have on multiple reproductive processes. In this regard, APS has been linked to a pro-inflammatory endometrial state, defective decidualization, altered trophoblastic invasion, and placental damage [17,90]. Several authors point to inflammation-mediated changes as primarily responsible for endometrial damage and impaired implantation, with APAs directly interfering with proper uterine decidualization [91,92]. 

Indeed, the nature of APS, as a pro-inflammatory and autoimmune disease, is evidenced, for instance, by studies that observed, in relation to this condition, an overexpression of certain chemokine genes (CCL20, CXCL3, CX3CL1, CXCL5, CXCL2, and CXCL1), involved in the recruitment, chemotaxis, and proliferation of mononuclear cells and/or granulocytes [93]. Similarly, other research reported a marked in vitro production of INF-γ in APS patients, suggesting a Th1 polarization. The authors propose that this elevated secretion could be related to APS-associated fetal losses, taking into account the Th2 responses commonly described in healthy pregnancies [94]. 

Interestingly, another recent study found a differential distribution of NK subsets according to the presence of different APAs, with an increase in the cytotoxic activity of these cells. Based on these results, APAs appeared to be able to promote antibody-dependent cellular cytotoxicity, suggesting NK cells as candidates for APA-related obstetric complications [95]. Other studies in this regard have also found significant alterations in T-cell populations, which are drastically reduced in women diagnosed with APS. These cells, in particular Tregs, play an essential role, among others, in the establishment of maternal–fetal tolerance and endometrial priming, with the authors proposing a relationship between these low cell counts and situations of insufficient decidualization [15]. 

In fact, one of the latest investigations into the reproductive consequences of APS has identified aberrant decidual microenvironments in women suffering from this disease, describing alterations at the cellular and molecular level that profoundly affect endometrial priming [96]. With this research, it becomes evident that APS has a significant effect on the endometrium, altering, at the same time, cell populations, cytokine production, and gene expression, all towards a pro-inflammatory condition, detrimental for both decidualization and pregnancy.

However, not only can these disturbances alter endometrial functionality, but even direct interactions between APAs and decidua have been observed by in vivo studies [91]. One of the first pieces of evidence in this regard comes from a 1990 study, which showed that mice treated with IgG APAs displayed decidual necrosis associated with intravascular deposits of IgG and fibrin [97]. In addition, APAs show a similar affinity for the placenta, triggering, upon binding, the recruitment of the complement system, neutrophil infiltration, and local release of pro-inflammatory cytokines such as TNF-α [91]. 

All of these have been linked to APS-related thrombosis and abnormal placentation, potentially leading to the obstetric syndromes observed in these patients [91,98]. Likewise, APAs are also capable of blocking endometrial angiogenesis, hindering the initial vascularization processes and preventing proper decidualization. This effect may be explained by the inhibition of specific angiogenic products, such as vascular endothelial growth factor (VEGF), by APAs [91,99]. 

In conclusion, it can be stated that APS, due to its autoimmune nature, promotes a situation of immune hyperactivity and pro-inflammation, completely disruptive within the endometrial environment. In addition to this alteration of decidualization and implantation, APS hinders the processes of vascularization and trophoblastic invasion, compromising placentation. This disease increases episodes of thrombosis in the already compromised placentas, leading to undesirable outcomes, like miscarriages and pre-eclampsia, commonly faced by these patients [17,91].

To date, management by a multidisciplinary team is strongly recommended for women diagnosed with APS, with the aim of preventing inflammatory damage and thrombotic complications as much as possible. In order to inhibit the placental binding of APAs, low-dose aspirin is currently being considered for pre-conception in APA carriers with no history of thromboembolism or obstetric complications [100,101]. This medication, beyond preventing platelet aggregation, may counteract APA-mediated hypercoagulability. On the other hand, for those patients who have suffered previous miscarriages, the use of low-molecular-weight heparin together with aspirin, after the confirmation of pregnancy, is often an option in the context of ART [101,102,103]. 

Regarding the immunological hyperactivity that this condition entails, other studies have evaluated the introduction of hydroxychloroquine and glucocorticoids to these discussed anticoagulant treatments. The use of these drugs in patients with APS is based on their anti-inflammatory properties by inhibiting the release of specific cytokines, modulating the immune response, impeding the process of antigen presentation, and preventing platelet aggregation and activation [104,105]. Likewise, the safety of these medications during pregnancy has been widely demonstrated [106]. 

Treating this disease from an immunological perspective seems to be the preferred option, given promising results for anti-inflammatory + anticoagulant combination therapy in patients with APS, improving their reproductive outcomes. In addition, the study of each case and the personalization of treatment, offering the most appropriate combination of currently accessible therapies (aspirin, heparin, glucocorticoids, or hydroxychloroquine) can contribute to significantly improving patients’ prospects [104]. Based on all that has been reviewed so far, the inflammatory aspect of APS emerges as evident, along with its direct impact on the endometrium and, therefore, on fertility, a route that both researchers and clinicians should follow in dealing with this condition.

### 3.2. Thyroid Dysfunction

Maternal thyroid dysfunction has been linked to reduced female fertility, a higher risk of fetal loss, and pregnancy complications, in part due to the existence of autoantibodies and sexual dysfunction induced by hypothyroidism [13,107]. There is growing evidence indicating that the presence of TPO antibodies, which are the most prevalent in this form of autoimmunity, is closely linked to undesired reproductive outcomes, as miscarriage, preterm birth, and the onset of thyroid disorders during pregnancy [108].

The precise mechanism through which antithyroid antibodies may influence pregnancy development remains unknown, with this phenomenon occurring even in the absence of thyroid dysfunction [109]. However, it should be noted that the presence of these and other autoantibodies, as in the case of APS, is already a sign of hyperactivity and pro-inflammation, which, although systemic, can have a direct and local impact on fertility-related organs [14].

Indeed, in the case of endometrium, a significant increase in T cells has been recorded in women with autoimmune thyroid diseases that tested positive for anti-thyroid antibodies and exhibited reduced fertility, compared to controls, who are negative for these autoantibodies. In addition, increased levels of pro-inflammatory cytokines, like IFN-γ, and a reduction in anti-inflammatory cytokines, such as IL-10 and IL-4, have been linked to patients with this kind of autoimmunity [110]. 

Alterations at the cellular and molecular level are, therefore, occurring at the endometrium of these women, which, potentially, could, as in the case of other diseases discussed in this review, alter uterine receptivity, immune tolerance, and implantation [13,111]. Beyond these endometrial alterations, it has also been highlighted that thyroid hormones are essential for oocyte maturation and for the menstrual cycle, suggesting several possible mechanisms by which thyroid dysfunction may alter fertility outcomes [109].

Interestingly, certain studies have indicated a correlation between APS and thyroid autoimmunity, with some pointing to shared pathophysiological mechanisms and genetic factors [112,113]. This relationship suggests that the assessment of women experiencing RPL should incorporate the screening for APAs, with particular attention paid to patients with thyroid autoimmunity and miscarriages after euploid embryo transfers [114]. 

Currently, TSH determination is part of all pregestational panel testing for patients undergoing ART, with the advice to treat thyroid dysfunction by administering levothyroxine. However, further studies including careful patient selection, euploid embryo transfers, and autoimmune screening (TPO, APAs, etc.) are needed to fully clarify the association between thyroid dysfunction and fertility, as well as to develop possible case-specific treatments, always taking into account the immunological perspective of the disease [114,115].

### 3.3. Pancreatic Autoimmunity

Diabetes is a chronic metabolic condition marked by increased blood glucose levels, traditionally categorized into Type 1 and Type 2. However, this classification does not cover all metabolic disorders associated with impaired insulin secretion or function. For example, we can highlight the existence of latent adult-onset diabetic autoimmunity (LADA), which represents the most prevalent form of adult-onset autoimmune diabetes [14,116].

LADA is characterized by the presence of diabetes-associated autoantibodies and a clear need for insulin therapy [116]. Interestingly, a high frequency of thyroid and gastric autoimmunity has been described among LADA patients [117], pointing to a genetic association between these autoimmune endocrine diseases. Additionally, diabetes-associated autoantibodies (DAAs) may appear even years before the diagnosis of LADA, indicating the possible existence of a prolonged pre-diabetic autoimmune period in these patients [116]. 

**Figure 3:**
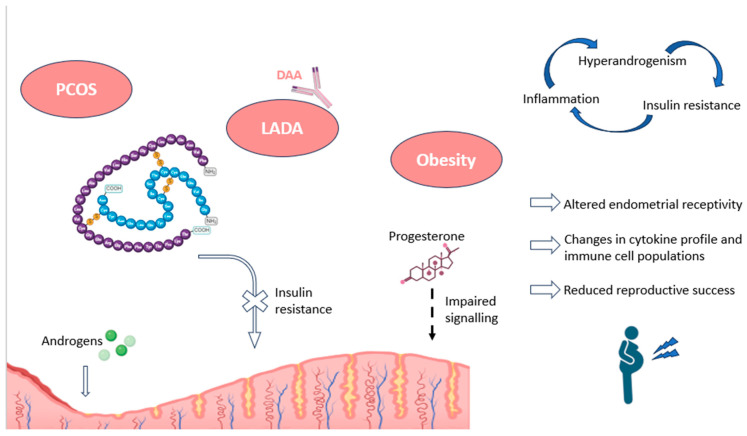
Impact of pancreatic autoimmunity on endometrium, along with associated pathologies, as PCOS and obesity. The endometrial effects and the main hormonal alterations recorded in these conditions are shown, including an increased endometrial presence of androgens, and a resistance to insulin signaling, as well as progesterone unresponsiveness. The figure also depicts the relationship between the most characteristic features of these pathologies (inflammation, insulin resistance, and hyperandrogenism). DAA: diabetes-associated autoantibodies; LADA: latent adult-onset diabetic autoimmunity; PCOS: polycystic ovary syndrome. Original, created with Biorender.com (accessed on 22 May 2024).

In the reproductive field, it has been found that pancreatic autoimmunity can cause considerable alterations in endometrial receptivity, through mechanisms involving obesity and inflammation (Figure 3). In particular, some authors have described impairments in progesterone signaling, necessary for proper decidualization, in patients suffering from hyperinsulinemia and insulin resistance. Interestingly, both metabolic conditions are present in obesity and polycystic ovary syndrome (PCOS), disorders with a high association with subfertility [17].

An example of the involvement that pancreatic hormones actually have in decidualization can be found in insulin receptor substrate 2 (IRS2), which is directly regulated by the progesterone receptor during decidualization. Adequate insulin signaling through this receptor appears necessary in order to control uterine gene expression and glucose utilization during endometrial priming. Alterations in glucose transporter expression and glucose uptake have been reported in cases of IRS2 down-regulation, demonstrating the importance of insulin signaling in the decidualization process and, consequently, in successful implantation and pregnancy [118].

Indeed, hyperinsulinemia and insulin resistance have been shown to repress markers of endometrial responsiveness, such as IGF-1 receptors, and lead to situations of hyperandrogenism within the uterine microenvironment [119]. Some of these abnormalities in endometrial receptivity can be observed in obese patients, who show significant transcriptomic differences, associated with steroid hormone signaling, compared to non-obese women. The alterations have been recorded in genes mainly involved in chemokine, cytokine, and immune system activity, among others [120]. Consequently, many authors suggest that these disturbances may contribute to poorer reproductive outcomes, such as lower implantation rates, associated with obesity [17].

Similar situations have been reported in PCOS patients, who show impaired progesterone-mediated decidualization and alterations in cytokine profiles and immune cell migration. In this regard, a higher percentage of CD68+ macrophages, M2 CD163+ macrophages, CD8+ T cells, and other related immune populations have been observed in women with PCOS compared to controls. These alterations point to a pro-inflammatory environment in the endometrium of these women, negative for implantation and pregnancy (Figure 3). The authors also found an association between these endometrial immune cells and insulin resistance, as further evidence of the importance of pancreatic hormones in decidualization [121]. 

Overall, endometrial insulin resistance, along with impaired glucose transport and utilization, leads to environments of chronic inflammation, changes in uterine blood flow, aberrations in gene expression patterns within the endometrium, and irregularities in uterine immune populations. All these alterations can negatively affect endometrial receptivity and enhance undesirable reproductive outcomes, such as RPL or RIF (Figure 3) [17]. Moreover, in the case of pregnancy, the continued exposure of the embryo to a high glucose environment could lead to glucotoxicity and the dysregulation of protein glycosylation, potentially causing recurrent miscarriages in these patients [122]. Furthermore, alterations in Treg cells, which have been described in patients with LADA, could complicate the process of maternal–fetal tolerance and potentially increase the risk of the disruption of early placentation, leading to similar undesired reproductive outcomes [116,123]. 

All these endometrial alterations and reproductive consequences have led researchers to investigate the appropriate management of these patients, especially in the context of ART. In this regard, our group demonstrated a significant improvement in LBR after metabolic control in patients with pancreatic autoimmunity, showing that proper diagnosis and treatment could have a positive impact on reproductive outcomes [116]. These results may serve to reconsider the current use of most tests for functional glucose disturbance, which are normally only offered during pregnancy but not in the preconception period, when, as discussed, insulin resistance can greatly affect endometrial receptivity [17,116].

In this sense, immune or metabolic routine screening can be useful for some subsets of patients suffering “silent” immune or metabolic disorders. To date, only fasting glucose is tested for glycemic disorders, although this determination may be insufficient in some cases. According to our results, patients with RPL or RIF of unknown etiology diagnosed with thyroid autoimmune disorders, family history of diabetes, and impaired insulin response after an oral glucose tolerance test (OGTT) could be considered as candidates for further specific autoimmune tests to determine potential DAA [116].

If positive, their treatment with metformin/insulin administration (the current accessible therapies for glucose metabolism disorders, also used in cases of obesity or PCOS) could considerably improve their reproductive prospects. This treatment is supposed to reduce the chronic inflammatory environment within the endometrium and possibly restore the adequate hormonal responsiveness for proper decidualization, among other consequences. According to the current literature, the delicate balance between steroid hormones, pancreatic hormones, endometrial immune activity, and uterine receptivity is a complex issue that must be taken into account in the management of ART patients [116]. 

### 3.4. Celiac Disease

Celiac disease (CD) is currently acknowledged as an immune-mediated systemic disorder linked to the consumption of dietary gluten in genetically predisposed individuals. It is associated with a range of symptoms, with some of them being mild or even subclinical. This broad spectrum of clinical presentations contributes to the significant delay in the diagnosis and underdiagnosis of CD [124,125]. With respect to the implication this disorder has on fertility, it has been reported that undiagnosed CD can be associated with negative reproductive outcomes, including RPL intrauterine growth restriction or pre-term birth [126,127]. 

However, despite this evidence, no consensus has been reached to consider infertility in the group of conditions associated with CD, with some controversy in the existing literature [125]. Nevertheless, the immune alterations associated with this disease seem to be evident. Indeed, imbalances between the pro-inflammatory signals of CD4+ T cells and the anti-inflammatory response of Treg cells have been described in these patients, leading to the secretion of cytokines such as IL-17, which strongly promote inflammation [128].

**Figure 4:**
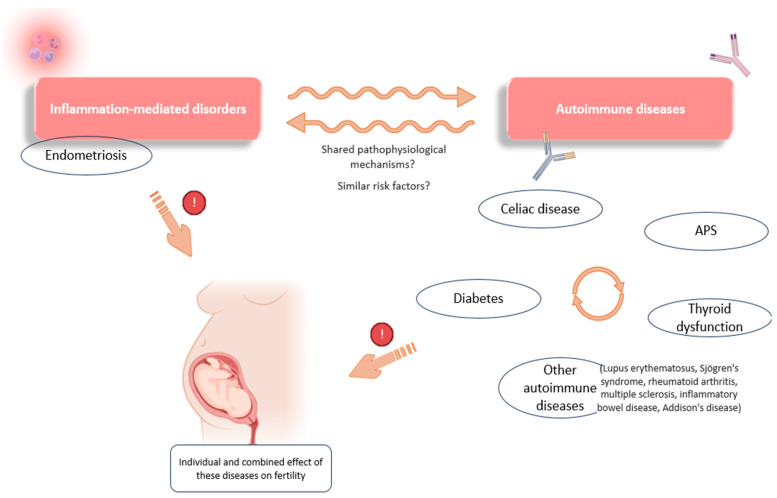
Some of the associations observed between inflammation-mediated conditions and autoimmunity. Links have been reported between the occurrence of different autoimmune conditions (celiac disease, diabetes, APS, and thyroid dysfunction). There are also associations between the development of endometriosis and certain immune diseases (thyroid dysfunction, celiac disease, lupus erythematosus, etc.), which point to possible shared pathophysiological mechanisms and a combined effect on reproductive function. APS: antiphospholipid syndrome. Original, created with Biorender.com and SMART.servier.com (accessed on 22 May 2024).

This situation of immune over-activation and chronic pro-inflammation is, as already discussed, a common feature of autoimmune disorders that, per se, has a negative impact on reproduction. Moreover, a link appears to exist between the development of CD and other autoimmune disorders, pointing to the altered systemic immune behavior in these women (Figure 4). For example, shared pathogenic mechanisms have been described for CD and type 1 diabetes, both conditions being linked to closely related high-risk human lymphocyte antigens (HLA-DR-DQ) [128].

Associations have also been observed between CD, thyroid dysfunctions, and APS, suggesting similar genetic and pathophysiological factors in these autoimmune disorders, as evidenced, for example, by the increased prevalence of certain APAs in patients diagnosed with thyroid autoimmunity [129]. Further evidence of this relationship was shown by a meta-analysis investigating the effect of a gluten-free diet in patients with Hashimoto’s thyroiditis. These authors found a positive effect of gluten deprivation on thyroid function and its related inflammation, demonstrating a link between these two disorders [130]. In addition, thyroid-related conditions and gastrointestinal diseases are frequently observed in patients with endometriosis, suggesting a common link not only between autoimmune diseases, but also with any inflammation-mediated disorder (Figure 4) [22,48,49].

Beyond these relationships with other infertility risk conditions, CD appears to be able to individually affect reproductive outcomes by a direct effect of anti-transglutaminase antibodies on the endometrium, being detrimental to angiogenesis, thus affecting placentation and trophoblast invasion, with these antibodies also being able to bind to trophoblast cells, further impacting these processes [131]. Therefore, and according to some other authors, the autoantibodies found in celiac disease seem to have direct adverse effects on placental function, while compromising other aspects such as nutrient transfer, decidualization, and fetal growth [132].

Consistent with these statements, our group reported better reproductive outcomes after a gluten-free diet in celiac patients compared to a normal diet, suggesting that CD is an immune-mediated disease whose early diagnosis and dietary treatment may improve patients’ reproductive success. It is, therefore, recommended that, after diagnosis, patients should start a gluten-free diet as a safe and effective therapeutic approach [125]. 

However, with respect to asymptomatic people, some controversy exists, with the mass screening for CD being debatable. Currently, the available knowledge does not permit the provision of specific recommendations regarding the general screening of celiac disease (CD) in women experiencing recurrent reproductive failure. Further prospective studies are still needed to consider the immune-mediated mechanisms that this disease and its autoantibodies may have on reproduction and to properly assess the beneficial effect of a gluten-free diet on reproductive outcomes [125].

## 4. Discussion

Immune activity and inflammatory processes are sometimes overlooked when it comes to reproductive success, as most views focus on other aspects, such as embryo quality. However, fertility works in a bidirectional way, and, nowadays, both clinicians and researchers are becoming aware that the maternal side, the endometrium, requires similar attention in order to achieve a healthy pregnancy [4,133]. 

Specifically, decidualization, or endometrial preparation, has been shown to be essential in reproduction, with numerous hormonal and cellular factors combining to completely transform the endometrium until the most suitable environment is created to welcome the incoming blastocyst. This process has a real impact on reproduction, with many undesirable outcomes faced by patients with defective endometrial priming. Moreover, the complexity of decidualization, involving numerous hormonal, cellular, and molecular factors that must act in an appropriate and finely controlled manner, is further evidence of the relevance of this event in fertility [5,133].

In view of this, it is not surprising that certain diseases, with a demonstrated direct impact on the endometrium, are gaining the attention of experts in the field. Among them, endometriosis, adenomyosis, and chronic endometritis stand out. These reproductive diseases have been recognized and studied for years, although they are now being examined from a new perspective. In fact, the numerous advances in reproductive immunology over the last few decades have helped to discern some of the mechanisms by which these conditions affect fertility, through their local effect on the endometrium [17]. 

Accordingly, important alterations in immune cell populations, in cytokine secretion, and even in gene expression have been described in women suffering from these diseases, pointing to the maternal immune microenvironment as a key factor for correct placentation and implantation, both events being altered in these patients. One of the most worrisome and characteristic features of these disorders is the establishment, within the endometrium, of a state of chronic inflammation, which completely alters the uterine environment and impacts decidualization, thus reducing reproductive success [16,17,19]. 

Specifically, altered steroid hormone signaling along with increases in endometrial pro-inflammatory macrophages, T cells, and cytotoxic NK cells, among other imbalances, have been described in endometriosis patients. These immunological alterations seem to contribute to the symptomology of this reproductive illness, enhancing pain and the development of endometriotic lesions, and decreasing reproductive success [19,21,26]. 

Similar immune imbalances are observed in women with adenomyosis, with associated endocrine disorders and low progesterone responsiveness. These situations lead these patients to face scenarios of persistent inflammation and decidualization dysfunction, which can significantly alter their fertility outcomes [61,64]. However, one of the most obvious cases of endometrial disruption is chronic endometritis. The different pathogens associated with the development of this disease strongly enhance immune responses that alter the healthy immune cellular microenvironment and delay endometrial priming [12,72]. 

Nevertheless, these are not the only conditions that alter fertility in this respect. Indeed, systemic disorders of an autoimmune nature also seem to have an impact on fertility in general, and on the endometrium in particular. Women who suffer from them show systemic immunological maladaptations which, when studied at the endometrial level, demonstrate their potential to alter their reproductive prospects. Examples are APS, thyroid dysfunction, LADA, or CD. Many authors have shown that these systemic diseases can affect the endometrial immune response, promoting pro-inflammatory states and impairing the normal progression of the menstrual cycle [14,116,125].

In fact, direct antibody-mediated endometrial/decidual damage has been observed in cases of APS, being completely detrimental to normal angiogenesis and proper placentation. A pro-inflammatory environment is also created around these already damaged placentas, with an increase in cytotoxic NK cells and inflammatory T-cell subsets [90,91,92]. These pro-inflammatory environments are also observed in the endometrium of women suffering from thyroid dysfunctions, a pathology that can alter fertility through many different mechanisms, directly and indirectly related to antibody-associated damage [13,114]. 

Of particular concern is the situation of unresponsiveness to progesterone in patients diagnosed with pancreatic autoimmunity. These scenarios lead to altered endometrial priming and negative reproductive outcomes, also observed in other conditions with shared pathophysiological mechanisms, such as obesity and PCOS [17,116]. Finally, celiac disease appears to affect reproductive outcomes by enhancing an environment of chronic inflammation and, by a direct effect of antibodies on the endometrium, impairing angiogenesis and affecting placentation and trophoblast invasion [131].

All these conditions, both local (endometriosis, adenomyosis, and chronic endometritis) and systemic (APS, thyroid dysfunction, LADA, and CD) have been related to each other, sharing similar pathophysiological pathways and endometrial consequences, with a predominance of chronic inflammatory processes that can alter, among others, progesterone responsiveness and, thus, implantation and proper placentation. The maternal immune system has been shown to be actively involved in all these events, and some of the mechanisms related to decreased fertility in these patients are trying to be fully discerned by researchers [17,130].

In fact, there are still many unknowns to be resolved in this regard, and the same applies when it comes to the diagnosis and treatment of some of these conditions. Although management protocols and therapeutic options already exist for some of them, as in the case of antibiotic regimens for chronic endometritis or a gluten-free diet for patients with CD, many doubts and controversies still arise, especially in the context of ART [17,125]. 

At present, continued research into these diseases, based on an immunological perspective and an understanding of their inflammatory nature, is the way forward for experts in the study of these conditions [17,116]. Research focusing on the immune imbalances described in these pathologies, as well as on progesterone unresponsiveness and antibody-mediated damage, are the main lines to follow in order to answer the current unknowns about these illnesses, while seeking targeted treatments for all of them. 

The full understanding of their reproductive consequences and the development of immunological treatments for them, as is already the case with some therapies for endometriosis [41], will undoubtedly improve our knowledge of the relationship between these diseases, immunology, and fertility, while increasing patients’ reproductive success.

## 5. Conclusions

Certain fertility-associated diseases, widely studied in terms of their reproductive impact, are now being investigated from a new perspective, from the sphere of immunology, offering new explanations as to their pathophysiology, as well as innovative treatment perspectives. In this regard, important endometrial alterations at all biological levels have been described in association with endometriosis, adenomyosis, and chronic endometritis. All of these lead to chronic inflammatory scenarios that alter key immunological events such as maternal–fetal tolerance or endometrial hormone responsiveness, negatively affecting decidualization and the progression of pregnancy.

Treatment of these diseases, mainly with hormonal therapies and NSAIDs in the case of endometriosis and adenomyosis, as well as the use of oral antibiotics after the confirmation of EC by the CD138 marker, is the current recommended course of action in order to improve the reproductive outcomes of patients. However, new immunological therapies continue to be developed to treat these pathologies with an increasingly targeted approach.

Similarly, systemic diseases closely related to the reproductive field are showing a direct impact at the endometrial level. The autoimmunity implicit in pathologies such as APS, thyroid dysfunction, pancreatic autoimmunity, or celiac disease can directly affect the endometrial immune microenvironment. In this way, such conditions promote situations of progesterone resistance, as well as antibody-mediated damage, which drastically alter decidualization and placentation.

A combined treatment with anticoagulant and anti-inflammatory medication for patients diagnosed with APS, thyroid supplementation (levothyroxine) in the case of dysfunction, and metformin/insulin treatment for diabetes and its associated immunity, as well as a gluten-free diet for CD patients are the lines of action in any reproductive treatment involving these types of autoimmunity. Understanding these diseases from an inflammatory and immunological perspective will allow us to find more specific and personalized treatments to improve the immune and, consequently, reproductive status of patients. 

## References

[1] Setti A.S., Braga D.P.d.A.F., Vingris L., Iaconelli A., Borges E. (2022). Improved embryonic development and utilization rates with EmbryoScope: A within-subject comparison versus a benchtop incubator. Zygote.

[2] Barnes J., Brendel M., Gao V.R., Rajendran S., Kim J., Li Q., Malmsten J.E., Sierra J.T., Zisimopoulos P., Sigaras A. (2023). A non-invasive artificial intelligence approach for the prediction of human blastocyst ploidy: A retrospective model development and validation study. Lancet Digit. Health.

[3] Alegre L., Del Gallego R., Arrones S., Hernández P., Muñoz M., Meseguer M. (2019). Novel noninvasive embryo selection algorithm combining time-lapse morphokinetics and oxidative status of the spent embryo culture medium. Fertil. Steril..

[4] Ng S.-W., Norwitz G.A., Pavlicev M., Tilburgs T., Simón C., Norwitz E.R. (2020). Endometrial Decidualization: The Primary Driver of Pregnancy Health. Int. J. Mol. Sci..

[5] Dunk C., Kwan M., Hazan A., Walker S., Wright J.K., Harris L.K., Jones R.L., Keating S., Kingdom J.C.P., Whittle W. (2019). Failure of Decidualization and Maternal Immune Tolerance Underlies Uterovascular Resistance in Intra Uterine Growth Restriction. Front. Endocrinol..

[6] Garrido-Gómez T., Castillo-Marco N., Cordero T., Simón C. (2022). Decidualization resistance in the origin of preeclampsia. Am. J. Obstet. Gynecol..

[7] Ticconi C., Di Simone N., Campagnolo L., Fazleabas A. (2021). Clinical consequences of defective decidualization. Tissue Cell.

[8] Diaz-Gimeno P., Sebastian-Leon P., Sanchez-Reyes J.M., Spath K., Aleman A., Vidal C., Devesa-Peiro A., Labarta E., Sánchez-Ribas I., Ferrando M. (2022). Identifying and optimizing human endometrial gene expression signatures for endometrial dating. Hum. Reprod..

[9] Piccinni M.-P., Raghupathy R., Saito S., Szekeres-Bartho J. (2021). Cytokines, Hormones and Cellular Regulatory Mechanisms Favoring Successful Reproduction. Front. Immunol..

[10] Kuroda K., Nakagawa K., Horikawa T., Moriyama A., Ojiro Y., Takamizawa S., Ochiai A., Matsumura Y., Ikemoto Y., Yamaguchi K. (2021). Increasing number of implantation failures and pregnancy losses associated with elevated Th1/Th2 cell ratio. Am. J. Reprod. Immunol..

[11] Wang Q., Zhang J., Wang F. (2023). A Study of the Predictive Value of Treg and Th1/Th2 Cytokines on Pregnancy Outcome in Patients with Recurrent Pregnancy Loss. Altern. Ther. Health Med..

[12] Pirtea P., Cicinelli E., De Nola R., de Ziegler D., Ayoubi J.M. (2021). Endometrial causes of recurrent pregnancy losses: Endometriosis, adenomyosis, and chronic endometritis. Fertil. Steril..

[13] Tańska K., Gietka-Czernel M., Glinicki P., Kozakowski J. (2023). Thyroid autoimmunity and its negative impact on female fertility and maternal pregnancy outcomes. Front. Endocrinol..

[14] Marder W., Littlejohn E.A., Somers E.C. (2016). Pregnancy and autoimmune connective tissue diseases. Best Prac. Res. Clin. Rheumatol..

[15] Krivonos M.I., Khizroeva J.K., Zainulina M.S., Eremeeva D.R., Selkov S.A., Chugunova A., Bitsadze V.O., Arslanbekova M., Sultangadzhieva K. (2020). The role of lymphocytic cells in infertility and reproductive failures in women with antiphospholipid antibodies. J. Matern.-Fetal Neonatal Med..

[16] Bourdon M., Santulli P., Jeljeli M., Vannuccini S., Marcellin L., Doridot L., Petraglia F., Batteux F., Chapron C. (2021). Immunological changes associated with adenomyosis: A systematic review. Hum. Reprod. Update.

[17] Franasiak J.M., Alecsandru D., Forman E.J., Gemmell L.C., Goldberg J.M., Llarena N., Margolis C., Laven J., Schoenmakers S., Seli E. (2021). A review of the pathophysiology of recurrent implantation failure. Fertil. Steril..

[18] Giudice L.C. (2010). Endometriosis. NEJM.

[19] Vallvé-Juanico J., Houshdaran S., Giudice L.C. (2019). The endometrial immune environment of women with endometriosis. Hum. Reprod. Update.

[20] Burney R.O., Giudice L.C. (2012). Pathogenesis and pathophysiology of endometriosis. Fertil. Steril..

[21] Houshdaran S., Nezhat C.R., Vo K.C., Zelenko Z., Irwin J.C., Giudice L.C. (2016). Aberrant Endometrial DNA Methylome and Associated Gene Expression in Women with Endometriosis. Biol. Reprod..

[22] Tamaresis J.S., Irwin J.C., Goldfien G.A., Rabban J.T., Burney R.O., Nezhat C., DePaolo L.V., Giudice L.C. (2014). Molecular Classification of Endometriosis and Disease Stage Using High-Dimensional Genomic Data. Endocrinology.

[23] Ahn S.H., Khalaj K., Young S.L., Lessey B.A., Koti M., Tayade C. (2016). Immune-inflammation gene signatures in endometriosis patients. Fertil. Steril..

[24] Takebayashi A., Kimura F., Kishi Y., Ishida M., Takahashi A., Yamanaka A., Wu D., Zheng L., Takahashi K., Suginami H. (2014). Subpopulations of Macrophages within Eutopic Endometrium of Endometriosis Patients. Am. J. Reprod. Immunol..

[25] Mor G., Cardenas I., Abrahams V., Guller S. (2011). Inflammation and pregnancy: The role of the immune system at the implantation site. Ann. N. Y. Acad Sci..

[26] Vallvé-Juanico J., Santamaria X., Vo K.C., Houshdaran S., Giudice L.C. (2019). Macrophages display proinflammatory phenotypes in the eutopic endometrium of women with endometriosis with relevance to an infectious etiology of the disease. Fertil. Steril..

[27] Laganà A.S., Salmeri F.M., Ban Frangež H., Ghezzi F., Vrtačnik-Bokal E., Granese R. (2019). Evaluation of M1 and M2 macrophages in ovarian endometriomas from women affected by endometriosis at different stages of the disease. Gynecol. Endocrinol..

[28] Bulletti C., Coccia M.E., Battistoni S., Borini A. (2010). Endometriosis and infertility. J. Assist. Reprod. Genet..

[29] Miller R.J., Jung H., Bhangoo S.K., White F.A. (2009). Cytokine and Chemokine Regulation of Sensory Neuron Function. Handb. Exp. Pharmacol..

[30] Ji R.-R., Chamessian A., Zhang Y.-Q. (2016). Pain regulation by non-neuronal cells and inflammation. Science.

[31] Schulke L., Berbic M., Manconi F., Tokushige N., Markham R., Fraser I.S. (2009). Dendritic cell populations in the eutopic and ectopic endometrium of women with endometriosis. Hum. Reprod..

[32] Giuliani E., Parkin K.L., Lessey B.A., Young S.L., Fazleabas A.T. (2014). Characterization of Uterine NK Cells in Women with Infertility or Recurrent Pregnancy Loss and Associated Endometriosis. Am. J. Reprod. Immunol..

[33] Diaz-Hernandez I., Alecsandru D., Garcia-Velasco J.A., Dominguez F. (2021). Uterine natural killer cells: From foe to friend in reproduction. Hum. Reprod. Update.

[34] Nowak I., Płoski R., Barcz E., Dziunycz P., Kamiński P., Kostrzewa G., Milewski Ł., Roszkowski P.I., Senitzer D., Malejczyk J. (2015). KIR2DS5 in the presence of HLA-C C2 protects against endometriosis. Immunogenetics..

[35] Kitawaki J., Xu B., Ishihara H., Fukui M., Hasegawa G., Nakamura N., Mizuno S., Ohta M., Obayashi H., Honjo H. (2007). Association of Killer Cell Immunoglobulin-like Receptor Genotypes with Susceptibility to Endometriosis. Am. J. Reprod. Immunol..

[36] de Barros I.B.L., Malvezzi H., Gueuvoghlanian-Silva B.Y., Piccinato C.A., Rizzo L.V., Podgaec S. (2017). What do we know about regulatory T cells and endometriosis? A systematic review. J. Reprod. Immunol..

[37] Podgaec S., Dias Junior J.A., Chapron C., Oliveira RMd Baracat E.C., Abrão M.S. (2010). Th1 and Th2 immune responses related to pelvic endometriosis. Rev. Assoc. Med. Bras..

[38] Gogacz M., Winkler I., Bojarska-Junak A., Tabarkiewicz J., Semczuk A., Rechberger T., Adamiak A. (2016). Increased percentage of Th17 cells in peritoneal fluid is associated with severity of endometriosis. J. Reprod. Immunol..

[39] Galgani M., Insabato L., Calì G., Della Gatta A.N., Mirra P., Papaccio F., Santopaolo M., Alviggi C., Mollo A., Strina I. (2015). Regulatory T cells, inflammation, and endoplasmic reticulum stress in women with defective endometrial receptivity. Fertil. Steril..

[40] Wang W.J., Hao C.F., Qu Q.L., Wang X., Qiu L.H., Lin Q.D. (2010). The deregulation of regulatory T cells on interleukin-17-producing T helper cells in patients with unexplained early recurrent miscarriage. Hum. Reprod..

[41] Saunders P.T.K., Horne A.W. (2021). Endometriosis: Etiology, pathobiology, and therapeutic prospects. Cell.

[42] Rana N., Braun D.P., House R., Gebel H., Rotman C., Dmowski W.P. (1996). Basal and stimulated secretion of cytokines by peritoneal macrophages in women with endometriosis. Fertil. Steril..

[43] Bruner-Tran K.L., Herington J.L., Duleba A.J., Taylor H.S., Osteen K.G. (2013). Medical management of endometriosis: Emerging evidence linking inflammation to disease pathophysiology. Minerva Ginecol..

[44] Burney R.O., Talbi S., Hamilton A.E., Vo K.C., Nyegaard M., Nezhat C.R., Lessey B.A., Giudice L.C. (2007). Gene Expression Analysis of Endometrium Reveals Progesterone Resistance and Candidate Susceptibility Genes in Women with Endometriosis. Endocrinology.

[45] Lessey B.A., Palomino W.A., Apparao K.B.C., Young S.L., Lininger R.A. (2006). Estrogen receptor-alpha (ER-alpha) and defects in uterine receptivity in women. Reprod. Biol. Endocrinol..

[46] Bishop L.A., Gunn J., Jahandideh S., Devine K., Decherney A.H., Hill M.J. (2021). Endometriosis does not impact live-birth rates in frozen embryo transfers of euploid blastocysts. Fertil. Steril..

[47] Paffoni A., Casalechi M., De Ziegler D., Cicinelli E., Somigliana E., Viganò P., Vitagliano A. (2024). Live Birth After Oocyte Donation In Vitro Fertilization Cycles in Women With Endometriosis. JAMA Netw. Open..

[48] Shigesi N., Kvaskoff M., Kirtley S., Feng Q., Fang H., Knight J.C., Missmer S.A., Rahmioglu N., Zondervan K.T., Becker C.M. (2019). The association between endometriosis and autoimmune diseases: A systematic review and meta-analysis. Hum. Reprod. Update.

[49] Korošec S., Riemma G., Šalamun V., Rutar A.F., Laganà A.S., Chiantera V., De Franciscis P., Frangež H.B. (2024). Coexistence of Endometriosis and Thyroid Autoimmunity in Infertile Women: Impact on in vitro Fertilization and Reproductive Outcomes. Gynecol. Obstet. Investig..

[50] Chapron C., Marcellin L., Borghese B., Santulli P. (2019). Rethinking mechanisms, diagnosis and management of endometriosis. Nat Rev. Endocrinol..

[51] França P.R.d.C., Lontra A.C.P., Fernandes P.D. (2022). Endometriosis: A Disease with Few Direct Treatment Options. Molecules.

[52] Vercellini P., Viganò P., Somigliana E., Fedele L. (2013). Endometriosis: Pathogenesis and treatment. Nat. Rev. Endocrinol..

[53] Bulun S.E., Yilmaz B.D., Sison C., Miyazaki K., Bernardi L., Liu S., Kohlmeier A., Yin P., Milad M., Wei J. (2019). Endometriosis. Endocr. Rev..

[54] Cheong Y.C. (2002). IL-1, IL-6 and TNF-alpha concentrations in the peritoneal fluid of women with pelvic adhesions. Hum. Reprod..

[55] Lu D., Song H., Shi G. (2013). Anti-TNF-α treatment for pelvic pain associated with endometriosis. Cochrane Database Syst. Rev..

[56] Rižner T.L., Penning T.M. (2020). Aldo-keto reductase 1C3—Assessment as a new target for the treatment of endometriosis. Pharmacol. Res..

[57] Greaves E., Horne A.W., Jerina H., Mikolajczak M., Hilferty L., Mitchell R., Fleetwood-Walker S.M., Saunders P.T.K. (2017). EP2 receptor antagonism reduces peripheral and central hyperalgesia in a preclinical mouse model of endometriosis. Sci. Rep..

[58] Zhao Y., Gong P., Chen Y., Nwachukwu J.C., Srinivasan S., Ko C., Bagchi M.K., Taylor R.N., Korach K.S., Nettles K.W. (2015). Dual suppression of estrogenic and inflammatory activities for targeting of endometriosis. Sci. Transl. Med..

[59] Sekulovski N., Whorton A.E., Tanaka T., Hirota Y., Shi M., MacLean J.A., de Mola J.R.L., Groesch K., Diaz-Sylvester P., Wilson T. (2020). Niclosamide suppresses macrophage-induced inflammation in endometriosis. Biol. Reprod..

[60] Etrusco A., Barra F., Chiantera V., Ferrero S., Bogliolo S., Evangelisti G., Oral E., Pastore M., Izzotti A., Venezia R. (2023). Current Medical Therapy for Adenomyosis: From Bench to Bedside. Drugs.

[61] Zhihong N., Yun F., Pinggui Z., Sulian Z., Zhang A. (2016). Cytokine Profiling in the Eutopic Endometrium of Adenomyosis During the Implantation Window After Ovarian Stimulation. Reprod. Sci..

[62] Tremellen K.P., Russell P. (2012). The distribution of immune cells and macrophages in the endometrium of women with recurrent reproductive failure. II: Adenomyosis and macrophages. J. Reprod. Immunol..

[63] Gui T., Chen C., Zhang Z., Tang W., Qian R., Ma X., Cao P., Wan G. (2014). The disturbance of TH17-Treg cell balance in adenomyosis. Fertil. Steril..

[64] Kobayashi H. (2023). Endometrial Inflammation and Impaired Spontaneous Decidualization: Insights into the Pathogenesis of Adenomyosis. Int. J. Environ. Res. Public Health.

[65] Kaunitz A.M. (2000). Menstruation: Choosing whether … and when. Contraception.

[66] Juárez-Barber E., Cozzolino M., Corachán A., Alecsandru D., Pellicer N., Pellicer A., Ferrero H. (2023). Adjustment of progesterone administration after endometrial transcriptomic analysis does not improve reproductive outcomes in women with adenomyosis. RBMO.

[67] Vannuccini S., Luisi S., Tosti C., Sorbi F., Petraglia F. (2018). Role of medical therapy in the management of uterine adenomyosis. Fertil. Steril..

[68] Maia H., Haddad C., Pinheiro N., Casoy J. (2013). The effect of oral contraceptives on aromatase and Cox-2 expression in the endometrium of patients with idiopathic menorrhagia or adenomyosis. Int. J. Womens Health.

[69] Cozzolino M., Pellicer N., Galliano D., Pellicer A. (2023). Pituitary suppression with GnRH agonists before ART may be insufficient to treat women with severe adenomyosis. RBMO.

[70] Che X., Wang J., He J., Guo X., Li T., Zhang X. (2020). The new application of mifepristone in the relief of adenomyosis-caused dysmenorrhea. Int. J. Med. Sci..

[71] Ferrero S., Remorgida V., Maganza C., Venturini P.L., Salvatore S., Papaleo E., Candiani M., Maggiore U.L.R. (2014). Aromatase and endometriosis: Estrogens play a role. Ann. N. Y. Acad. Sci..

[72] Kitaya K., Takeuchi T., Mizuta S., Matsubayashi H., Ishikawa T. (2018). Endometritis: New time, new concepts. Fertil. Steril..

[73] McQueen D.B., Bernardi L.A., Stephenson M.D. (2014). Chronic endometritis in women with recurrent early pregnancy loss and/or fetal demise. Fertil. Steril..

[74] Cicinelli E., De Ziegler D., Nicoletti R., Colafiglio G., Saliani N., Resta L., Rizzi D., De Vito D. (2008). Chronic endometritis: Correlation among hysteroscopic, histologic, and bacteriologic findings in a prospective trial with 2190 consecutive office hysteroscopies. Fertil. Steril..

[75] Kitaya K., Yasuo T. (2010). Aberrant expression of selectin E, CXCL1, and CXCL13 in chronic endometritis. Mod. Pathol..

[76] Tortorella C., Piazzolla G., Matteo M., Pinto V., Tinelli R., Sabbà C., Fanelli M., Cicinelli E. (2014). Interleukin-6, interleukin-1β, and tumor necrosis factor α in menstrual effluents as biomarkers of chronic endometritis. Fertil. Steril..

[77] Matteo M., Cicinelli E., Greco P., Massenzio F., Baldini D., Falagario T., Rosenberg P., Castellana L., Specchia G., Liso A. (2009). ORIGINAL ARTICLE: Abnormal Pattern of Lymphocyte Subpopulations in the Endometrium of Infertile Women with Chronic Endometritis. Am. J. Reprod. Immunol..

[78] Salama S.A., Kamel M.W., Diaz-Arrastia C.R., Xu X., Veenstra T.D., Salih S., Botting S.K., Kumar R. (2009). Effect of Tumor Necrosis Factor-α on Estrogen Metabolism and Endometrial Cells: Potential Physiological and Pathological Relevance. J. Clin. Endocrinol. Metab..

[79] Kitaya K., Yasuo T. (2011). Immunohistochemistrical and Clinicopathological Characterization of Chronic Endometritis. Am. J. Reprod. Immunol..

[80] Di Pietro C., Cicinelli E., Guglielmino M.R., Ragusa M., Farina M., Palumbo M.A., Cianci A. (2013). Altered Transcriptional Regulation of Cytokines, Growth Factors, and Apoptotic Proteins in the Endometrium of Infertile Women with Chronic Endometritis. Am. J. Reprod. Immunol..

[81] Wu D., Kimura F., Zheng L., Ishida M., Niwa Y., Hirata K., Takebayashi A., Takashima A., Takahashi K., Kushima R. (2017). Chronic endometritis modifies decidualization in human endometrial stromal cells. Reprod. Biol. Endocrinol..

[82] Bayer-Garner I.B., Korourian S. (2001). Plasma Cells in Chronic Endometritis are Easily Identified When Stained with Syndecan-1. Mod. Pathol..

[83] Guo L., Gu F., Tan J., Luo L., Gao J., Zhou C. (2020). Multiple endometrial polyps is associated with higher risk of chronic endometritis in reproductive-aged women. J. Obstet. Gynaecol. Res..

[84] Peng J., Guo J., Zeng Z., Liang X., Zeng H., Li M. (2022). Endometrial polyp is associated with a higher prevalence of chronic endometritis in infertile women. Int. J. Gynecol. Obstet..

[85] Kuroda K., Takamizawa S., Motoyama H., Tsutsumi R., Sugiyama R., Nakagawa K., Sugiyama R., Kuribayashi Y. (2021). Analysis of the therapeutic effects of hysteroscopic polypectomy with and without doxycycline treatment on chronic endometritis with endometrial polyps. Am. J. Reprod. Immunol..

[86] Cicinelli E., Matteo M., Tinelli R., Lepera A., Alfonso R., Indraccolo U., Marrocchella S., Greco P., Resta L. (2014). Prevalence of chronic endometritis in repeated unexplained implantation failure and the IVF success rate after antibiotic therapy. Hum. Reprod..

[87] Kim C.J., Romero R., Chaemsaithong P., Kim J.-S. (2015). Chronic inflammation of the placenta: Definition, classification, pathogenesis, and clinical significance. Am. J. Obstet. Gynecol..

[88] Ghidini A., Salafia C.M. (2005). Histologic placental lesions in women with recurrent preterm delivery. Acta Obstet. Gynecol. Scand..

[89] Pignatelli P., Ettorre E., Menichelli D., Pani A., Violi F., Pastori D. (2020). Seronegative antiphospholipid syndrome: Refining the value of “non-criteria” antibodies for diagnosis and clinical management. Haematologica.

[90] Petri M. (2020). Antiphospholipid syndrome. Transl. Res..

[91] Rodrigues V.O., Soligo A., Pannain G.D. (2019). Antiphospholipid Antibody Syndrome and Infertility. Rev. Bras. Ginecol. Obstet..

[92] Núñez-Álvarez C.A., Cabiedes J. (2011). Mecanismos patogénicos de los anticuerpos antifosfolípidos. Reumatol. Clín..

[93] Hamid C., Norgate K., D’Cruz D.P., Khamashta M.A., Arno M., Pearson J.D., Frampton G., Murphy J.J. (2007). Anti- 2GPI-antibody-induced endothelial cell gene expression profiling reveals induction of novel pro-inflammatory genes potentially involved in primary antiphospholipid syndrome. Ann. Rheum. Dis..

[94] Visvanathan S., McNeil H.P. (1999). Cellular immunity to beta 2-glycoprotein-1 in patients with the antiphospholipid syndrome. J. Immunol..

[95] Manukyan G., Kriegova E., Slavik L., Mikulkova Z., Ulehlova J., Martirosyan A., Papajik T. (2023). Antiphospholipid antibody-mediated NK cell cytotoxicity. J. Reprod. Immunol..

[96] Lu C., Gao R., Qing P., Zeng X., Liao X., Cheng M., Qin L., Liu Y. (2024). Single-cell transcriptome analyses reveal disturbed decidual homoeostasis in obstetric antiphospholipid syndrome. Ann. Rheum. Dis..

[97] Branch D.W., Dudley D.J., Mitchell M.D., Creighton K.A., Abbott T.M., Hammond E.H., Daynes R.A. (1990). Immunoglobulin G fractions from patients with antiphospholipid antibodies cause fetal death in BALB/c mice: A model for autoimmune fetal loss. Am. J. Obstet. Gynecol..

[98] Shamonki J.M., Salmon J.E., Hyjek E., Baergen R.N. (2007). Excessive complement activation is associated with placental injury in patients with antiphospholipid antibodies. Am. J. Obstet. Gynecol..

[99] Chighizola C.B., de Jesus G.R., Branch D.W. (2016). The hidden world of anti-phospholipid antibodies and female infertility: A literature appraisal. Autoimmun. Rev..

[100] Latino J.O., Udry S., Wingeyer S.P., Romero D.F., Micone P., de Larrañaga G. (2018). What is the best time to assess the antiphospholipid antibodies (aPL) profile to better predict the obstetric outcome in antiphospholipid syndrome (APS) patients?. Immunol. Res..

[101] Franasiak J.M., Scott R.T. (2017). Contribution of immunology to implantation failure of euploid embryos. Fertil. Steril..

[102] Tektonidou M.G., Andreoli L., Limper M., Amoura Z., Cervera R., Costedoat-Chalumeau N., Cuadrado M.J., Dörner T., Ferrer-Oliveras R., Hambly K. (2019). EULAR recommendations for the management of antiphospholipid syndrome in adults. Ann. Rheum. Dis..

[103] Grygiel-Górniak B., Mazurkiewicz Ł. (2023). Positive antiphospholipid antibodies: Observation or treatment?. J. Thromb. Thrombolysis.

[104] Ye S.-L., Gu X.-K., Tao L.-Y., Cong J.-M., Wang Y.-Q. (2017). Efficacy of Different Treatment Regimens for Antiphospholipid Syndrome-related Recurrent Spontaneous Abortion. Chin. Med. J..

[105] Arachchillage D.J., Laffan M., Pericleous C. (2023). Hydroxychloroquine as an Immunomodulatory and Antithrombotic Treatment in Antiphospholipid Syndrome. Int. J. Mol. Sci..

[106] Flint J., Panchal S., Hurrell A., van de Venne M., Gayed M., Schreiber K., Arthanari S., Cunningham J., Flanders L., Moore L. (2016). BSR and BHPR guideline on prescribing drugs in pregnancy and breastfeeding—Part I: Standard and biologic disease modifying anti-rheumatic drugs and corticosteroids: Table 1. Rheumatology.

[107] Lee Y.L., Ng H.P., Lau K.S., Liu W.M., O W.S., Yeung W.S., Kung A.W. (2009). Increased fetal abortion rate in autoimmune thyroid disease is related to circulating TPO autoantibodies in an autoimmune thyroiditis animal model. Fertil. Steril..

[108] Dhillon-Smith R.K., Coomarasamy A. (2020). TPO antibody positivity and adverse pregnancy outcomes. Best Pract. Res. Clin. Endocrinol. Metab..

[109] Deroux A., Dumestre-Perard C., Dunand-Faure C., Bouillet L., Hoffmann P. (2016). Female Infertility and Serum Auto-antibodies: A Systematic Review. Clin. Rev. Allergy Immunol..

[110] Stewart-Akers A.M., Krasnow J.S., Brekosky J., Deloia J.A. (2011). Endometrial Leukocytes Are Altered Numerically and Functionally in Women with Implantation Defects. Am. J. Reprod. Immunol..

[111] Matalon S.T., Blank M., Ornoy A., Shoenfeld Y. (2003). The Association Between Anti-Thyroid Antibodies and Pregnancy Loss. Am. J. Reprod. Immunol..

[112] Ong S.G., Choy C.H. (2015). Autoimmune thyroid disease in a cohort of Malaysian SLE patients: Frequency, clinical and immunological associations. Lupus.

[113] Kim N.Y., Cho H.J., Kim H.Y., Yang K.M., Ahn H.K., Thornton S., Park J.C., Beaman K., Gilman-Sachs A., Kwak-Kim J. (2011). Thyroid autoimmunity and its association with cellular and humoral immunity in women with reproductive failures. Am. J. Reprod. Immunol..

[114] Alecsandru D., Garcia Velasco J.A. (2020). Levothyroxine and thyroid peroxidase antibodies in women with recurrent pregnancy loss. Fertil. Steril..

[115] De Groot L., Abalovich M., Alexander E.K., Amino N., Barbour L., Cobin R.H., Eastman C.J., Lazarus J.H., Luton D., Mandel S.J. (2012). Management of Thyroid Dysfunction during Pregnancy and Postpartum: An Endocrine Society Clinical Practice Guideline. J. Clin. Endocrinol. Metab..

[116] Alecsandru D., Barrio A., Andia V., Cruz E., Aparicio P., Serna J., Cruz M., Pellicer A., Garcia-Velasco J.A. (2018). Pancreatic autoimmunity: An unknown etiology on patients with assisted reproductive techniques (ART)-recurrent reproductive failure. PLoS ONE.

[117] Pipi E. (2014). Distinct clinical and laboratory characteristics of latent autoimmune diabetes in adults in relation to type 1 and type 2 diabetes mellitus. World J. Diabetes.

[118] Neff A.M., Yu J., Taylor R.N., Bagchi I.C., Bagchi M.K. (2020). Insulin Signaling Via Progesterone-Regulated Insulin Receptor Substrate 2 is Critical for Human Uterine Decidualization. Endocrinology.

[119] Schulte M.M.B., Tsai J.-H., Moley K.H. (2015). Obesity and PCOS: The Effect of Metabolic Derangements on Endometrial Receptivity at the Time of Implantation. Reprod. Sci..

[120] Comstock I.A., Diaz-Gimeno P., Cabanillas S., Bellver J., Sebastian-Leon P., Shah M., Schutt A., Valdes C.T., Ruiz-Alonso M., Valbuena D. (2017). Does an increased body mass index affect endometrial gene expression patterns in infertile patients? A functional genomics analysis. Fertil. Steril..

[121] Liu S., Hong L., Mo M., Xiao S., Chen C., Li Y., Lian R., Wang X., Cai S., Diao L. (2021). Evaluation of endometrial immune status of polycystic ovary syndrome. J. Reprod. Immunol..

[122] Sun X., Feng Y., Ma Q., Wang Y., Ma F. (2023). Protein glycosylation: Bridging maternal–fetal crosstalk during embryo implantation. Biol. Reprod..

[123] Liuzzo G., Biasucci L.M., Trotta G., Brugaletta S., Pinnelli M., Digianuario G., Rizzello V., Rebuzzi A.G., Rumi C., Maseri A. (2007). Unusual CD4+CD28nullT Lymphocytes and Recurrence of Acute Coronary Events. J. Am. Coll. Cardiol..

[124] Catassi C., Rätsch I.-M., Fabiani E., Rossini M., Coppa G., Giorgi P., Bordicchia F., Candela F. (1994). Coeliac disease in the year 2000: Exploring the iceberg. Lancet.

[125] Alecsandru D., Lopez-Palacios N., Castano M., Aparicio P., Garcia-Velasco J.A., Nunez C. (2020). Exploring undiagnosed celiac disease in women with recurrent reproductive failure: The gluten-free diet could improve reproductive outcomes. Am. J. Reprod. Immunol..

[126] Gasbarrini A., Torre E.S., Trivellini C., De Carolis S., Caruso A., Gasbarrini G. (2000). Recurrent spontaneous abortion and intrauterine fetal growth retardation as symptoms of coeliac disease. Lancet.

[127] Casella G., Orfanotti G., Giacomantonio L., Di Bella C., Crisafulli V., Villanacci V., Baldini V., Bassotti G. (2016). Celiac disease and obstetrical-gynecological contribution. Gastroenterol. Hepatol. Bed Bench.

[128] Goodwin G. (2019). Type 1 Diabetes Mellitus and Celiac Disease: Distinct Autoimmune Disorders That Share Common Pathogenic Mechanisms. Horm. Res. Paediatr..

[129] Dong A.C., Morgan J., Kane M., Stagnaro-Green A., Stephenson M.D. (2020). Subclinical hypothyroidism and thyroid autoimmunity in recurrent pregnancy loss: A systematic review and meta-analysis. Fertil. Steril..

[130] Piticchio T., Frasca F., Malandrino P., Trimboli P., Carrubba N., Tumminia A., Vinciguerra F., Frittitta L. (2023). Effect of gluten-free diet on autoimmune thyroiditis progression in patients with no symptoms or histology of celiac disease: A meta-analysis. Front. Endocrinol..

[131] Tersigni C., Castellani R., de Waure C., Fattorossi A., De Spirito M., Gasbarrini A., Scambia G., Di Simone N. (2014). Celiac disease and reproductive disorders: Meta-analysis of epidemiologic associations and potential pathogenic mechanisms. Hum. Reprod. Update.

[132] Özgör B., Selimoğlu M.A. (2009). Coeliac disease and reproductive disorders. Scand. J. Gastroenterol..

[133] Cuadrado-Torroglosa I., García-Velasco J.A., Alecsandru D. (2023). New insights into decidualization: Immunological and genetic factors. Curr. Opin. Obstet. Gynecol..

